# Improvement in children’s fine motor skills following a computerized typing intervention

**DOI:** 10.1016/j.humov.2017.10.013

**Published:** 2017-12

**Authors:** Hannah L. McGlashan, Caroline C.V. Blanchard, Nicole J. Sycamore, Rachel Lee, Blandine French, Nicholas P. Holmes

**Affiliations:** School of Psychology, University of Nottingham, University Park, Nottingham NG7 2RD, UK

**Keywords:** Dysgraphia, Typing intervention, Manual dexterity, Fine motor skills, MABC-2

## Abstract

Children spend a large proportion of their school day engaged in tasks that require manual dexterity. If children experience difficulties with their manual dexterity skills it can have a consequential effect on their academic achievement. The first aim of this paper was to explore whether an online interactive typing intervention could improve children’s scores on a standardised measure of manual dexterity. The second aim was to implement a serial reaction time tapping task as an index of children's finger movement learning, and to see whether performance on this task would improve after the intervention. Seventy-eight typically developing children aged between 8 and 10 were tested at their school on the pre-intervention Movement Assessment Battery for Children (2nd edition; MABC-2) and tapping tasks. Twenty-eight of these children volunteered to be randomly allocated to the intervention or control group. Children in the intervention group had a choice of two online games to play at home over a period of four weeks, while the children in the control group were not given these games to play. The intervention and control groups were then re-tested on the MABC-2 manual dexterity and the tapping task. Children in the intervention group significantly improved their manual dexterity scores in the MABC-2 compared to the control group. On average, all children learnt the tapping sequence, however, there were no group differences and no effect of the intervention on the tapping task. These results have important implications for implementing a freely available, easy to administer, fun and interactive intervention to help children improve their manual dexterity skills.

## 1 Introduction

Daily activities for children require a variety of motor skills, which are developed and refined through practice (Ungerleider, Doyon, & Karni, 2002). This includes balance, coordination, fine and gross motor skills. Fine manual skills are essential for children at a school-aged level, and problems with these skills can affect children in diverse ways (McHale & Cermak, 1992). For instance, fine manual skills determine handwriting performance including speed and legibility (Exner, 1989, Simner, 1982). Handwriting performance can, in turn, determine a child’s quality and quantity of learning and achievement in the classroom, and consequently have an influence on a child’s self-esteem and motivation (Cermak and Henderson, 1990, Levine et al., 1987).

Fine motor difficulties can affect a child’s academic performance because the child may attend to the mechanical aspects of written work instead of concentrating on the content of the work (May-Benson, Ingolia, & Koomar, 2002). Moreover, poor fine motor control is responsible for incorrect size or placement of letters, and inadequate pencil grip, which may result in slow, jerky writing (Simner, 1982). Illegible handwriting can prevent the development of higher-order skills such as spelling and story writing (Feder & Majnemer, 2007). Children who have problems with fine motor skills are often fatigued by hand written school work, and often take much longer to complete their work (May-Benson et al., 2002). The consequences of fine motor difficulties resulting in poor handwriting, or dysgraphia – with a prevalence in school-aged children ranging from 10 to 30% (Karlsdottir & Stefansson, 2002), include a tendency towards lower achievement in mathematics, lower verbal IQ, and increased attentional difficulties (Sandler et al., 1992). Furthermore, children in junior school can spend up to 60% of their school day completing tasks that involve fine motor skills, and 80% of their time completing drawing and writing based tasks (McHale & Cermak, 1992). Due to the extent that impairments in fine motor skills can impact academic achievement in children, it is crucial that schools implement interventions for children with notable difficulties.

Previous research into motor interventions generally falls into one of two different approaches. The first approach is 'process-oriented', and focuses on the suspected underlying process of the motor deficit such as sensory functions, memory, attention, planning, and formulating motor programs (Laszlo and Bairstow, 1985, Laszlo et al., 1988). The second approach is 'task-oriented', and involves remediation through the practice of a specific task that results in skill generalization (Schmidt, 1975). Task-orientated approaches focus on tasks that are causing the child difficulties (Henderson and Sugden, 1992, Revie and Larkin, 1993, Wright and Sugden, 1998). For the most effective intervention, occupational therapists and physiotherapists often adopt an eclectic approach, which combines elements of both process- and task-oriented methods. There is strong evidence to support both approaches, but the reason for their success is not clear (Sugden & Chambers, 2003). As the incidence of fine motor impairments is so high, it is important that other means of intervention or support are available, other than that from skilled professionals. Parents and teachers can also contribute to this intervention process, with research exploring developmental coordination disorder (DCD) finding that both teachers and parents can provide effective intervention for this motor condition, at home or at school respectively (Sugden & Chambers, 2003). Sugden and Chambers (2003) found that teachers and parents using a task-oriented intervention significantly improved scores on all sub-tests of the standardized Movement Assessment Battery for Children (2nd Edition; MABC-2) post intervention. Interestingly, although this intervention focused on one specific motor deficit, the children’s overall motor performance increased, implying an underlying, more general, motor process was affected.

Occupational therapists, teachers, and parents alike have suggested using a computer word-processor with a keyboard as a solution to fine-motor difficulties in handwriting (Niles-Campbell, Tam, Mays, & Skidmore, 2008). However, evidence to support this recommendation in typically developing children is lacking (Klein et al., 2008). One of the few studies that do provide evidence for the use of word-processing to improve fine motor skills in typically developing children found that an intervention using computer software significantly improved 7–8 year old children’s visual-motor skills (Chwirka, Gurney, & Burtner, 2002). The software used graphically presented hand and finger placement for each key, was self-paced, and had short lessons with visual reinforcers. Chwirka et al. (2002) commented that students were highly motivated to pursue this type of intervention because they enjoyed the use of a computer. The authors noted that keyboarding is not mechanically the same as handwriting, but there are similarities between the two activities, such that the practice of one may lead to improvement in the other (Chwirka et al., 2002).

The current study performed an intervention for improving fine motor skills. The first aim of this report was to test whether practicing typing skills with an interactive online game, similar to that used by Chwirka et al. (2002), could improve performance in children’s fine motor skills. It was hypothesized that children in the intervention group would score significantly higher on the manual dexterity sub-tasks of the MABC-2 after the intervention period, compared to children in the control group who were not expected to significantly improve their score. We also tested performance on an abstract visual-motor tapping task, based on well-studied finger movement sequence learning tasks (Clegg, DiGirolamo, & Keele, 1998). This task is closely related to the intervention (i.e., requires sequential finger movements), is highly controllable, and completely novel to all the children. It was used both as an additional measure to assess children’s fine motor skills in a way that is similar to the intervention, as well as to provide an index of children's finger movement learning skill. Therefore, the second aim of this study was to explore whether the performance on this tapping task improved after the intervention period. It was hypothesized that children in the intervention group would have a shorter reaction time and make fewer errors after the intervention period than children in the control group. It was also hypothesized that children would implicitly learn a finger movement sequence by having a shorter reaction time and perform fewer errors for a repeating sequence of movements compared to a random sequence of finger movements.

## 2 Method

### 2.1 Participants

All parents and children gave written, informed consent and assent, respectively. The experimental procedures were approved by the local ethical review committees at the University of Nottingham, and were in accordance with the Declaration of Helsinki (as of 2008).

Participants were recruited from an original sample of 78 children, recruited through their school in connection with a local DCD support group, and tested on the MABC-2 and tapping tasks. 65 children (24 males; mean ± SD age = 9.37 ± 0.73 years) were included in the sample for the tapping experiment, following exclusion of 13 participants determined by two criteria. The first criterion was for children to perform at least one correct tap in each set of eight trials per sequence, and the second was for children to have at least 62.5% correct responses (i.e., 5/8) in the pre-intervention test.

For the intervention phase, consent forms were sent out to all the 65 parents for their children to take part in the intervention, and 60 parents were also contacted by telephone. 28 consent forms were returned, allowing 28 children to participate in the post-intervention test. The intervention condition consisted of 12 children (of the 28), however 3 of these did not complete their intervention diary at home, and were excluded from the dataset, leaving 9 children who successfully completed the intervention (intervention group; 3 males; mean ± SD age = 9.41 ± 0.57 years). The remaining 16 children (of the 28) participated as controls and did not complete the intervention (control group; 7 males; mean ± SD age = 9.32 ± 0.64 years). After applying the two exclusion criteria, two children from each group were excluded; this left 7 children in the intervention group and 14 in the control group for analysis of the effect of the intervention on children performance on the tapping task (21 in total).

## 3 Measures

### 3.1 Serial reaction time measure

Finger tapping ability was assessed by a custom computerised manual dexterity tapping game – a version of the serial reaction time task (SRTT). This tapping game required the children to push a series of four keys with four different fingers of their dominant hand (index, middle, ring, and little fingers) on a keyboard in response to the indicated finger that displayed for 1s on a laptop screen. The task was divided into two conditions, random and sequence. The random condition contained a pseudorandomised sequence of finger stimuli, each cueing a required single keypress. Every repetition of 8 trials contained each finger twice, in two sub-blocks of 4 trials, in a random order within each sub-block. The sequence condition contained a regular and repeating sequence of fingers (in two sub-blocks of 4, e.g.: repetition 1 = 1-2-4-3, 4–1-3-2; repetition 2 = 1-2-4-3, 4-1-3-2…). Two practice blocks were first presented to participants. At first the task consisted of 12 repeats of each 8 trials sequence for each condition (random or sequence), but this proved too long for the children, as performance was generally poor in the last three repetitions (most of the excluded participants had performed the 12-repetition task), so it was reduced to 9 repetitions of 8 trials per condition (a total of 144 trials per child). For the participants who returned for the post-intervention retest, it was again reduced to 8 repetitions of 8 trials per condition (a total of 128 trials per child), because we had observed a worsening in performance after this point.

### 3.2 Motor performance measure

Motor performance was assessed using the standardized MABC-2 (Henderson, Sugden, & Barnett, 2007). The MABC-2 consists of eight tasks in three domains of movement, assessing manual dexterity (placing pegs, threading lace, drawing a trail), aiming and catching (catching a ball with two hands, throwing a beanbag onto a target), and static and dynamic balance (standing on a balance board, walking heel to toe, hopping between mats). All children completed the MABC-2 in the 7–10 year-old bracket (age band 2) before the intervention period and then the manual dexterity sub-tests were repeated after the intervention period for the intervention and control groups only.

### 3.3 Cognitive ability measure

A short version of the British Ability Scales 2nd edition (BAS-2) was used to assess educational achievement. The sub-tests used included a single word reading task where participants had to read a list of words from a sheet that progressively increase in difficulty. A verbal similarities test was used; this required children to explain how lists of three words were related or similar to each other. Finally, a matrices task was used to assess non-verbal ability and required children to select the correct item from a list of six, which completed a sequential pattern. These three sub-tests were combined to yield an overall measure of each child’s general cognitive ability (GCA).

### 3.4 Intervention task

Children in the intervention group were asked to perform an interactive online typing computer game at home over a period of four weeks. They had a choice of two games to play, Dance Mat Typing (BBC Bitesize, 2016) or Typing Chef (Sense-lang, 2014). Dance Mat Typing teaches children touch-typing skills by breaking down the letters on the keyboard into learnable chunks per level and teaching hand positioning by presenting letters and words on the screen for children to type. Typing Chef has the aim of improving children’s typing speed by presenting a word, which has to be copied within a certain time limit. The children were asked to play this game 5 times a week for around 10 min each time, based on recommendations from a meta-analysis of intervention studies (Pless & Carlsson, 2000). The children's consistency and progress was checked with the parents each week via email. The children were asked to complete a table showing which game they chose, the date they played the game, and if they had progressed through any levels. The games were made for children aged 8–10 years old and were designed to improve children’s hand eye coordination and manual dexterity.

### 3.5 Design and procedure

First all children completed the whole MABC-2, the short BAS-2, and the tapping task in a one-hour session during a school day. Children in the intervention group then completed the intervention typing games over the next weeks, while the control group did not practice the games during this period, but were informed about what children in the intervention group were doing. The children in both the control and intervention groups then performed the tapping task and manual dexterity sub-tests of the MABC-2 a second time at school after the -week intervention period.

## 4 Results

All descriptive data reported are means ± SE to three significant figures, unless otherwise stated.

### 4.1 Performance on the serial reaction time task

**Fig. 1:**
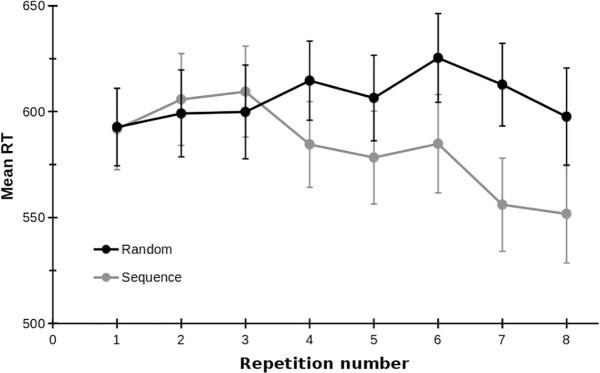
Mean ± SE reaction time (RT, ms) improves as a function of block number in the sequential condition (grey), but not in the random condition (black). Data from the first session of all 65 children who completed this task are included. The intervention had no significant effects on performance of the serial reaction time task.

To characterise learning in the two conditions (sequence and random, Fig. 1), a linear model was applied to each participant's data, estimating the steepness of the slope of reaction time (RT) as a function of repetition number. Correlations between repetition number and RT were calculated, and r-values were transformed to Z-scores (Fisher's transformation) to allow parametric statistical assessment of the strength of correlation between conditions and groups. Scores from the first repetition were excluded, as the participants would not have been able to distinguish the condition (sequence or random) at this point. Two 2 × 2 repeated measures ANOVAs with variables of time (pre, post) and condition (sequence, random) were conducted for the slope, and the Z-scores separately. There was a significant difference between sequence and random conditions on the slopes of RT across repetitions t(64) = 2.49, p = .015. Learning slopes for the sequence condition were −9.59 ± 2.51 ms/repetition compared to the random condition, which was +1.14 ± 3.31 ms/repetition. There was also a significant difference in the Z-scores (strength of correlation), t(64) = 3.45, p = .001. Z-scores for the sequence condition were greater (more negative; −0.150 ± 0.032) than for the random condition (0.027 ± 0.036).

### 4.2 Effects of the intervention on the serial reaction time task

Means and standard deviations for RT and proportion of correct responses before and after the intervention period for the control group and intervention group are shown in Table 1. Both the control and intervention groups' RT appears to decrease for both sequence and random conditions after the intervention period. Furthermore, while both groups' proportion of correct responses appears to increase for both conditions after the intervention period, analysis of these data revealed no significant effects on proportion correct. Additionally, in a MANOVA, including both RT and proportion correct, significant multivariate effects were driven by effects in RT. We therefore report only the RT analysis.

**Table 1:** Performance on the serial reaction time task.

Measure	Condition	Time	Group		Difference		
			Control (n = 7) (M ± SD)	Intervention (n = 14) (M ± SD)	*M*	*t*(19)	*p*
RT (ms)	Sequence	Before	551 ± 123	502 ± 259	49	0.608	0.550
		After	517 ± 104	445 ± 166	71	1.22	0.239
	Random	Before	588 ± 147	583 ± 144	5	0.081	0.936
		After	518 ± 98	477 ± 75	41	0.933	0.362
Correct (%)	Sequence	Before	84.5 ± 9.8	83.1 ± 9.5	1.4	0.301	0.766
		After	91.4 ± 7.2	83.3 ± 11.2	8.1	2.02	0.058
	Random	Before	81.0 ± 17.6	81.8 ± 8.5	0.8	0.108	0.916
		After	89.2 ± 8.5	87.2 ± 9.5	1.9	0.459	0.651

M: mean; SD: standard deviation; RT: reaction time; ms: milliseconds.

The ANOVA on the Z-scores (correlation strength) showed significant main effects of time, F(1,20) = 7.11, p = .015, η2 = 0.262, and condition, F(1,20) = 13.0, p = .002, η2 = 0.395. Z-scores were significantly greater (more negative) before (−0.145 ± 0.049) than after (0.025 ± 0.038) the intervention period, and were significantly greater for sequence (−0.185 ± 0.047) compared to random (0.066 ± 0.045) conditions. However, there were no significant interactions between condition and group, F(1,20) = 1.07, p = .313, time and group, F(1,20) = 0.343, p = .564, condition and time, F(1,20) = 0.344, p = .564, or condition, time, and group, F(1,20) = 1.49, p = .236.

The ANOVA on the slope data (learning rate) showed a significant main effect of condition, F(1,20) = 6.97, p = .016, η2 = 0.258. The slopes were greater for sequence (−8.83 ± 3.01 ms/repetition) than for random conditions (4.38 ± 3.35 ms/repetition). There was no significant effect of time, F(1,20) = 3.83, p = .064 or interactions between condition and group, F(1,20) = 0.93, p = .347, time and group, F(1,20) = 0.23, p = .64, condition and time, F(1,20) = 0.115, p = .738, or condition, time, and group, F(1,20) = 1.09, p = .310.

### 4.3 Effects of the intervention on MABC-2 scores

Eight out of the nine participants in the intervention group chose to play the Dance Mat Typing game, and spent an average of 158 min (SD = 46.6; range = 80–200 min) playing the game over a four-week period. One participant chose to play the Typing Chef game and spent only 15 min in total playing this over the four-week period, however the child’s data was still included in the analysis. There was no significant difference between groups in GCA (intervention group = 0.644 ± 0.327, control group = 0.850 ± 0.176; t(23) = −0.609, p = .549), or age (intervention group = 9.41 ± 0.155, control group = 9.32 ± 0.160; t(23) = 0.344, p = .734). Descriptive statistics of all MABC-2 manual dexterity scores pre- and post-intervention can be seen in Table 2. Table 2 also displays the difference in scores between pre- and post-intervention, with *t*-test statistics for each of these comparisons. We noticed that the statistical strength of the results changed depending on the score used in the MABC-2 sub-tests (i.e., item standard scores, component standard scores, and percentiles, see also French et al., submitted for publication), so we provide them all below. Across all measures, there were no significant differences between control and intervention groups in terms of their manual dexterity ability prior to the intervention period (Table 2).

**Table 2:** Performance on the MABC-2 manual dexterity tests (n = 25).

Measure	MABC-2 Subtest	Time	Group		Difference		
			Control (n = 9) M (SD)	Intervention (n = 16) M (SD)	M	t(23)	p
Raw	Pegboard dominant hand (s)	Before	25.9 (4.31)	26.4 (5.01)	−0.508	0.253	.803
		After	26.7 (3.40)	22.7 (2.60)	4.02	3.07	**.005**
	Pegboard non-dominant hand (s)	Before	31.5 (5.84)	27.0 (4.14)	4.50	1.94	.065
		After	29.8 (3.99)	24.9 (2.98)	4.91	3.19	**.004**
	Threading (s)	Before	20.8 (3.62)	21.4 (5.68)	−0.632	0.341	0.736
		After	22.3 (3.35)	19.6 (3.81)	−2.71	1.83	.082
	Drawing (errors)	Before	0.375 (0.500)	1.11 (1.62)	−0.736	1.71	.101
		After	0.50 (0.632)	0.00 (0.00)	0.500	2.35	**.028**
ISS	Pegboard (AU)	Before	9.56 (3.03)	10.1 (2.71)	−0.549	0.450	.657
		After	9.44 (2.56)	13.3 (1.58)	−3.90	4.13	**.001**
	Threading (AU)	Before	11.81 (2.34)	11.7 (3.08)	0.146	0.133	.895
		After	10.1 (3.14)	12.2 (2.05)	−2.10	1.79	.086
	Drawing (AU)	Before	9.25 (2.62)	7.67 (3.97)	1.58	1.20	.241
		After	8.94 (3.00)	11.1 (0.333)	2.17	2.15	**.043**
CS	Manual dexterity	Before	30.6 (5.99)	29.4 (3.64)	1.18	0.535	.597
		After	28.5 (5.81)	36.7 (2.74)	−8.17	3.95	**.001**
SS		Before	10.9 (3.01)	10.1 (1.69)	0.764	0.698	.492
		After	9.75 (2.77)	13.9 (1.83)	−4.14	3.95	**.001**
Percentile		Before	58.3 (28.7)	51.3 (21.3)	6.98	0.636	.531
		After	45.8 (28.2)	86.8 (11.7)	−41.0	4.14	**.001**
Logit		Before	0.229 (0.783)	0.027 (0.402)	0.202	0.718	.480
		After	−0.047 (0.711)	0.998 (0.530)	1.05	3.84	**.001**

M: mean; SD: standard deviation; s: seconds; ISS: item standard scores; AU: arbitrary units; CS: component scores; SS: standard scores.

### 4.4 Item standard scores

**Fig. 2:**
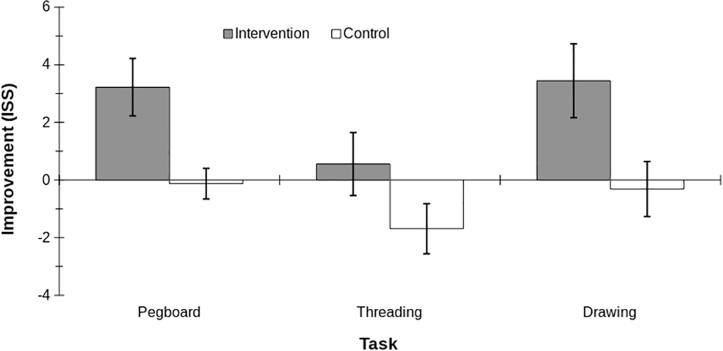
Touch-typing intervention improves children's performance on the pegboard, threading, and drawing tasks of the MABC-2. Data show mean ± SE changes in performance (improvement in item standard scores, ISS) in the post-intervention session compared to the pre-intervention session, for intervention (grey, n = 9) and control (white, n = 16) groups separately. dom. – dominant hand, non-dom. – non-dominant hand.

Item standard scores (ISS) were calculated for the pegboard, threading, and drawing tasks by converting their raw scores into standardized scores using the MABC-2 normative data tables. A 2 × 3 ANOVA, with within-participant variables of time (pre, post), and task (pegboard, threading, drawing), and the between-participants variable of group (intervention, control) was run on participant item standard scores. The results showed significant main effects for task, F(2,46) = 8.01, p < .001, and time, F(1,23) = 6.60, p = .017, and a significant interaction between group and time, F(1,23) = 22.2, p < .001. There were no significant interactions between task and group (F(2,46) = 1.53, p = .227), task and time (F(2,46) = 2.79, p = .072, or task, time, and group (F2,46) = 0.29, p = .754. Descriptive statistics in Table 2 and Fig. 2 show that whereas ISS for the intervention group increased post intervention by 3.22 ± 1.00 for the pegboard and 3.44 ± 1.28 for the drawing tasks, the control group’s ISS did not change significantly (pegboard = −0.125 ± 0.531; drawing = −0.313 ± 0.956). There was no significant difference between groups in the threading task.

### 4.5 Component standard scores and percentile scores

Component scores (CS) for manual dexterity were calculated by adding the three ISS together. These were converted into standard scores (SS), which are a normalized transformation of a distribution of raw scores, measured in standard deviation units and arguably the most suitable for research purposes as it gives the clearest indication of a child’s performance relative to their age group (Henderson et al., 2007). According to the MABC-2 manual, the percentiles indicate the percentage of children in the standardization sample who obtained a score less than or equal to a given raw score and are calculated from a norms table based on participants' CS. We have developed macros to automate the scoring of this task (Blanchard et al., 2017). Two 2 × 2 repeated measures ANOVA, with within-subject variables of time (pre, post) and the between-subjects variable of group (intervention, control) were run on participants' SS and percentiles. Percentile scores were first converted to proportions, then logit transformed so that the scores could be analysed with ANOVA. SS scores showed significant effects of time, F(1,23) = 7.32, p = .013, and a significant interaction between time and group, F(1,23) = 25.0, p < .001. The percentile data showed the same effects (time, F(1,23) = 7.00, p = .014; time × group, F(1,23) = 22.6, p < .001). The descriptive statistics (Table 2) revealed that the intervention group’s average CS improved by 7.22 ± 1.15 points, whereas the control group's average CS decreased by 2.13 ± 1.33 points. The average SS of the intervention group increased by 3.78 ± 0.66 (i.e., 1.26 standard deviations according to the norms table) post-intervention, compared to the control group, whose average score dropped by 1.13 ± 0.63 (0.377 standard deviations). Finally, the intervention group's average (untransformed) percentile score increased by 35.4 ± 6.78%, while the control group’s one decreased by 12.6 ± 6.13% percentage points. Following transformation, the average logit score increase was 0.971 ± 0.177 for the intervention, and decrease was −0.277 ± −0.169 for the control group. The ANOVA showed that there were significant main effects of time and a significant interaction between group and time for both the SS and the percentiles.

## 5 Discussion

The main aim of this report was to explore whether practicing typing skills with a real-world interactive online game could improve performance in children’s fine motor skills. The typing games shared the same specific practice of finger tapping with the tapping task, while they required similar, more general, motor processes – planning and control of movement – as those tested on the MABC-2. Our study investigated whether computer tapping games, like both task- and process-oriented methods, improve children's typing performance and their manual dexterity, respectively. According to our findings, offering children the opportunity to practice tapping skills, for instance by playing typing games, did not lead to improved tapping performance in the serial reaction time task, but did improve children's manual dexterity as a whole, giving more support to the process-oriented approach.

These results support our prediction that children in the intervention group would score significantly higher on the manual dexterity sub-tasks of the MABC-2 after the intervention period, compared to children in the control group who did not perform the typing game. Moreover, these findings confirm those of Chwirka et al. (2002), and support the notion that parents can be effective in implementing an intervention (Sugden & Chambers, 2003). In this case, practicing a typing game designed to improve children’s hand eye coordination and manual dexterity can generalize to improving scores on a standardized measure of manual dexterity. Importantly, children in the intervention group improved their manual dexterity standard score by more than 1 standard deviation (according to the MABC-2 norms tables) after the intervention period compared to the control group, which is a large improvement. One interpretation of this great improvement is that of a motivation effect. Having to perform these standardized tests a second time with no apparent reward could be a reason for the non-significant change in score in the control group. Likewise, an increase in motivation in the intervention group may be responsible for the very large improvement in scores. The children actively engaging in fun interactive games during the holiday period may have resulted in increased motivation to ‘show-off’ their newly developed skills. This is an important outcome if a motivating game can improve manual dexterity performance in children.

Although we found strong evidence that the intervention game improved children’s manual dexterity skills for the MABC-2, this improvement did not generalize to the task-oriented approach (i.e., the sequence-learning tapping task). We found effects of time and condition on participants' RT, suggesting that greater sequence learning is occurring in the first compared to the second tapping task session for both groups. But, there was no significant interaction to suggest a group difference across time. As there was less apparent sequence learning for both groups in the post intervention trial of the tapping task, it may be that complete learning of this task had already taken place in the pre-intervention period, or during the intervention period itself, for both groups, and therefore no further learning could take place. Both groups in the post intervention period scored almost perfectly across trials. An alternative explanation may be that learning an implicit movement sequence relies on distinctive cognitive processes that were not utilised while training in the touch-typing intervention.

Finally, our results supported our hypothesis that children would implicitly learn a finger movement sequence by having a shorter reaction time and perform fewer errors for a sequence compared to a random block of finger movements. The result for all 65 children – initially tested on the tapping task – showed a more negative slope and significantly more negative correlations for the sequence condition than the random condition. This means that children’s reaction times were getting shorter for the sequence condition as they progressed through blocks of repeated sequences.

The simulation model of Rumelhart and Norman (1982) can provide an explanation for the success of a typing intervention such as the one used for this study (Chwirka et al., 2002). They emphasised the importance of both internally elicited feedback of finger movements through tactile or proprioception, and external feedback through visual experience. When a child is learning to type in the initial stages of the game, they are relying on spatial visual and tactile inputs to correctly type a word. As they improve and progress through harder levels, children engage in cognitive processes more than motor learning, with less reliance on sensory feedback (Gordon, Casabona, & Soechting, 1994). It is suggested that learning to hand write and type both require perceptual feedback provided by vision, and by the tactile and proprioceptive systems, to improve fluency. Children who have difficulty with handwriting are shown to rely more on the visual motor integration process (Maeland, 1992, Tseng and Chow, 2000). Therefore, improving a child’s visual-motor skills could result in a secondary outcome of improving handwriting performance and consequently manual dexterity fluidity (Chwirka et al., 2002). This generalised improvement found within the intervention task described in this study supports the effects of a process-oriented task approach. The intervention involving different processes of the sensory system may have resulted in improvement in manual dexterity as a whole.

### 5.1 Limitation and future directions

One concern for the current study is that the spontaneous use of touch-typing training or exercises was not controlled for the control group, which limits the conclusion drawn from the results. Also, the relatively low participant number means the study’s findings should be taken with some caution. The intervention sample was restricted due to the time-scale, as participation occurred in the last week of term before the Christmas holiday, and the first week after. Furthermore, the researchers were limited by the available time that children could take out of their classes, and by the number of children within the selected age group that attended the school. The response rate for participating in the intervention was also low, with only 43% of parents returning consent forms. The pre-intervention tapping part of the experiment saw an even greater detriment to sample size, with participants excluded for poor performance. Due to the failure of three children to complete the intervention condition, there was an unequal and relatively small size for the intervention group. Future studies using such an intervention should, ideally, use larger and equal group sizes. However, as the effect sizes were very large for the MABC-2 manual dexterity scores, this demonstrates the potential efficacy of the intervention as a method of improving fine motor skills in children. A further improvement for future studies would be to re-test the other MABC-2 sub-tasks such as aiming and catching or balance as a control to see if the improvement is only in the manual dexterity domain, or generalises across movement domains. Further research is also required to apply this intervention to cohorts of children with dysgraphia, developmental coordination disorder, or other motor impairments to explore the clinical applications of this intervention. Another consideration would be to explore the improvements found with this process-oriented task, to a task-specific or task-oriented intervention.

## 6 Conclusion

This study has demonstrated the efficacy of a simple touch-typing intervention in improving the fine motor skills of children who do not have a specific motor deficit. This gives an exciting premise for future studies to explore this intervention in children with clinically diagnosed fine motor difficulty and in children with dysgraphia. With more resources and time, a larger future replication study would be beneficial to clarify the nature and size of the effects found in the current study, and to further explore the underlying processes responsible.
